# Efficacy and safety of a new intravenous immunoglobulin (Panzyga^®^) in chronic immune thrombocytopenia

**DOI:** 10.1111/tme.12573

**Published:** 2019-01-27

**Authors:** O. Arbach, A. B. Taumberger, S. Wietek, L. Cervinek, A. Salama

**Affiliations:** ^1^ Immunhaematology, Institut für Transfusionsmedizin, Universitätsmedizin Berlin Berlin Germany; ^2^ SPARK/Validation Fund, Berlin Health Innovations Berlin Germany; ^3^ Clinical Project Management, Octapharma Pharmazeutika Produktionsges.m.b.H. Vienna Austria; ^4^ Department of Internal Medicine, Hematology and Oncology University Hospital Brno Brno Czech Republic; ^5^ Klinik für Gynäkologie, Campus Virchow‐Klinikum Charite Universitätsmedizin Berlin Berlin Germany

**Keywords:** immune thrombocytopenia, intravenous immunoglobulin, ITP, IVIG, Panzyga^®^, platelet count, safety

## Abstract

**Objectives:**

To assess the efficacy and safety of intravenous immunoglobulin (IVIG) 10% (Panzyga^®^), a novel human normal IVIG 10%, in patients with chronic immune thrombocytopenia (ITP).

**Background:**

First‐line treatment options in ITP include IVIGs.

**Methods:**

In this prospective, open‐label, non‐controlled, multicentre, phase III study, patients received a daily dose of IVIG 10% (1 g kg^−1^ body weight) for two consecutive days. The primary end point was clinical response rate; secondary end points included alternate response definitions, time to response, response duration, platelet counts, regression of bleeding and safety.

**Results:**

Forty patients were enrolled (57·5% male, mean age 36·7 years); the full analysis set comprised 36 patients. A clinical response was seen for 29 of 36 patients (80·6%). Median time to response and response duration was 2 days and 14 days, respectively. IVIG 10% was well tolerated at a maximum infusion rate of 8 mg (kg min)^−1^ in all but one patient; adverse events were mainly mild to moderate in severity, and the most frequent was headache (42·5%).

**Conclusion:**

IVIG 10% is well tolerated even at a high infusion speed and induces a rapid platelet count increase, thus decreasing the bleeding rate and the severity of bleeding events.

Trial registry: http://ClinicalTrials.gov record: NCT01349790.

Immune thrombocytopenia (ITP) remains one of the best‐characterized human autoimmune diseases. It results from the production of autoantibodies to platelet antigens and, thereby, increased platelet destruction, leading to variable thrombocytopenia (Cines *et al.,* 2009; Lo & Deane, 2014; Audia *et al.,* 2017; Zufferey *et al.,* 2017).

Treatment is indicated at least in patients with significant bleeding or those at risk of bleeding (Provan *et al.,* 2010; Neunert *et al.,* 2011). Currently available drugs, including corticosteroids, other immunosuppressants and thrombopoietin receptor agonists, have limited efficacy (Salama, 2011; Michel, 2013) or the treatment effect cannot be predicted. Furthermore, the therapeutic effect of current treatments may be achieved only after several days or weeks, and many of the treated patients may not respond to the drug and/or may develop severe side effects, leading to discontinuation or switching of therapies (Salama, 2011; Neunert *et al.,* 2013).

In 1981, the administration of high‐dose intravenous immunoglobulins (IVIG) was reported to result in a relatively rapid increase in the platelet counts of children with ITP (Imbach *et al.,* 1981a; Imbach *et al.,* 1981b). Since then, IVIG administration has continued to increase (Hooper, 2008; Kerr *et al.,* 2014). Numerous studies have demonstrated that the administration of up to 1 g kg^−1^ IVIG is effective in 70–80% of patients with ITP. However, the vast majority of those studies were performed with IVIG 5% (Bussel & Pham, 1987; Newland *et al.,* 2001; Julia *et al.,* 2009; Dash *et al.,* 2014).

During the last decade, there has been a significant shift towards the use of IVIG 10% products. The benefits are shorter treatment time, provided equal safety and efficacy profiles are maintained, amongst others. IVIG 10% (Panzyga^®^; Octapharma AG, Wien, Österreich) is a novel high‐purity, glycine‐stabilised, liquid ready‐to‐use human immunoglobulin 10% (100 mg mL^−1^) IVIG product. It is manufactured using various precipitation and chromatography techniques for the harvesting and purification of immunoglobulin G. The process comprises three dedicated steps for pathogen safeguarding (solvent/detergent treatment, ion exchange chromatography and small‐pore nanofiltration at extreme pH) and results in a higher yield of IgG per litre plasma, a precious and limited resource. The primary objectives of this clinical trial were to assess the efficacy of IVIG 10% (Panzyga^®^) in the treatment of adult patients with chronic ITP and to study the safety of this high‐dose, new‐generation IVIG in a clinical setting.

## MATERIALS AND METHODS

### *Study design*

This was a prospective, open‐label, non‐controlled, multicentre, phase III study investigating the efficacy and safety of IVIG 10% (Panzyga^®^) in adults with primary chronic ITP (http://clinicaltrials.gov record: NCT01349790). The main inclusion criteria were: age 18–65 years (initially 18–80 years but reduced during the course of the study to implement a request from a regulatory authority), confirmed diagnosis of chronic primary ITP of ≥12 months' duration (diagnosed with threshold platelet count <100 × 10^9^ L^−1^; other causes of thrombocytopenia excluded through history, physical examination and blood test results) and platelet count ≤20 × 10^9^ L^−1^ with or without bleeding manifestations. The main exclusion criteria included: secondary or drug‐related ITP, Evans syndrome, treatment with IVIG or anti‐RhD within 3 months of enrolment, treatment with thrombopoietin receptor agonists or other platelet‐enhancing drugs (accepted therapies included long‐term stable corticosteroids, azathioprine, cyclophosphamide or attenuated androgen) within 3 weeks before enrolment, a history of unresponsiveness to previous IVIG or anti‐RhD immunoglobulin treatment, rituximab within 3 months of enrolment or splenectomy in the previous 4 weeks or planned splenectomy and known IgA deficiency with antibodies against IgA. All patients gave written informed consent.

The final analysis based on the 40 treated patients was completed using the defined end points because the US Food and Drug Administration consented to a 40‐patient cohort size (instead of targeted *n* = 95) due to favourable outcome and safety results.

### *Study medication*

Each patient enrolled in the study was to receive the study medication at a dose of 1 g kg^−1^ bodyweight, given daily for two consecutive days for a total of 2 g kg^−1^. The initial infusion rate was set to 0·01 mL (kg min)^−1^ [60 mg (kg h)^−1^], which was gradually increased to a maximum of 0·08 mL (kg min)^−1^ [480 mg (kg h)^−1^] if well tolerated by the patient.

### *Treatment outcomes*

The primary end point of the study was defined as the proportion of patients with an increased platelet count to ≥50 × 10^9^ L^−1^ at least once by day 8. Secondary end points included assessment of response rates using definitions set by the European Medicines Agency (EMA; Table S1, Supporting information) (Committee for Medicinal Products for Human Use, 2010). Bleeding severity was assessed by the investigator using a 6‐point verbal rating scale (Buchanan & Adix, 2002): 1 = none (no haemorrhage of any kind); 2 = minor [few petechiae (≤100 total) and/or ≤ 5 small bruises (≤3 cm diameter) but no mucosal bleeding]; 3 = mild [more than 100 petechiae and/or >5 large bruises (>3 cm diameter) but no mucosal bleeding]; 4 = moderate (overt mucosal bleeding, such as epistaxis, gum bleeding, oropharyngeal blood blisters, menorrhagia or gastrointestinal bleeding, not requiring immediate medical intervention); 5 = severe [mucosal bleeding or suspected internal haemorrhage (e.g. in the brain, lung, muscle or joint) requiring immediate medical intervention]; 6 = life‐threatening/fatal (documented intracranial haemorrhage or life‐threatening or fatal haemorrhage at any site).

The safety variables included the type and frequency of adverse events (AEs), post‐study‐related safety reports, drug overdose, drug interactions, drug misuse, medication errors, laboratory parameters (haematology, biochemistry, urinalysis, viral markers and direct Coombs' test), vital signs and physical examinations. Pregnancies were additionally monitored. AEs reported to the investigator were recorded at both scheduled and unscheduled study visits, and their severity (mild, moderate or severe) and seriousness (non‐serious or serious) were determined by the investigator. All AEs occurring or worsening following the initiation of study treatment were defined as treatment‐emergent AEs (TEAEs); their relationship to the study drug was assessed by the investigator and noted as probable, possible or unlikely.

### *Statistical analysis*

Several sets were defined for the analysis of outcomes. The safety analysis set (SAS) consisted of all patients enrolled in the study who received at least part of one dose of the study drug, whereas the full analysis set (FAS) consisted of all patients in the SAS who satisfied all major eligibility criteria and had at least one post‐baseline platelet concentration measurement [intention‐to‐treat (ITT) analysis]. The per‐protocol (PP) subset encompassed all patients in the FAS set excluding patients who had major protocol violations before the primary efficacy end point was reached, which may have had an impact on the primary outcome.

Evaluation of the primary end point was performed for the FAS (ITT analysis) and for the PP set (PP analysis). This primary analysis aimed to demonstrate that the response rate after administration of IVIG 10% (Panzyga^®^) was above a pre‐defined, constant reference value of 0·60.

The analyses were conducted using the statistical software SAS (version 9.1 or higher) (Cary (North Carolina),USA); for the pharmacokinetic analyses, Phoenix WinNonlin software (version 6.2 or later) (Certara Inc., Princeton, USA) was used.

### *Ethics*

The protocol was reviewed and approved by each study site's Independent Ethics Committee or Institutional Review Board before the start of the study. The study was conducted in accordance with the ethical principles that originate from the Declaration of Helsinki and the International Conference on Harmonization guideline E6: Good Clinical Practice. Patients provided written informed consent prior to study entry.

### *Subjects studied*

A total of 40 patients were enrolled into the study from 20 sites in Europe and India; 31 patients (77·5%) completed the study, and 9 patients (22·5%) withdrew prematurely after at least one administration of study medication due to investigator judgement (*n* = 3; patients at one study site required other drug treatment for ITP), withdrawal of consent (*n* = 2), death (*n* = 2; both unrelated to study treatment) and an AE (*n* = 1; worsening of thrombocytopenia considered unrelated to study treatment); one patient was lost to follow up. All 40 patients received at least one dose of study medication. A total of 77 infusions were administered; the majority of patients (37/40; 92·5%) received two infusions, and the remaining patients (3/40; 7·5%) received only one infusion.

The FAS comprised 36 patients (4 were excluded for lack of study eligibility), the PP set comprised 33, and the SAS comprised all 40 patients who were enrolled in the study; the baseline characteristics of this population are presented in Table 1. The SAS was 57·5% male, and had a mean age of 36·7 years (range 18–72), a mean bodyweight of 73·8 kg and a mean body mass index of 24·6 kg m^−2^. The mean duration of infusion at day 1 was 3·36 (±0·43) h and ranged from 3·00 to 5·50 h, whereas at day 2 it was 3·23 (±0·67) h, ranging from 0·62 to 5·08 h. At baseline, 13 patients (36·1%) in the FAS had no bleeding, 14 had minor (38·9%), 2 had mild (5·6%) and 7 had moderate (19·4%) bleeding overall. No patient in the FAS had a severe or life‐threatening/fatal bleed.

**Table 1:** Baseline characteristics and demographics of adult patients with chronic primary immune thrombocytopenia in the safety analysis set

Characteristic/demographic	*N* = 40
Age, years	
Mean ± SD	36·7 ± 15·34
Median (range)	32 (18–72)
Gender, *n* (%)	
Male	23 (57·5)
Female	17 (42·5)
Ethnicity, *n* (%)	
Caucasian	36 (90·0)
Asian	4 (10·0)
Bodyweight, kg	
Mean ± SD	73·8 ± 14·81
Median (range)	72 (52–110)
BMI	
Mean ± SD	24·6 ± 3·87
Median (range)	24 (19–33)
Median (range) alcohol consumption, units week^−1^	0 (0–40)
Smoking status, *n* (%)	
Non‐smoker	32 (80·0)
Ex‐smoker	5 (12·5)
Smoker	3 (7·5)
Median (range) ITP disease duration, months	47·2 (13–317)
Splenectomy, *n* (%)^1^	10 (25·0)

BMI, body mass index; ITP, immune thrombocytopenia; SD, standard deviation.

1 Four male and six female patients (mean age 39·2 years, mean bodyweight 77·4 kg) with no significant differences to overall study group underwent splenectomy.

## RESULTS

### *Efficacy*

#### Primary end point

A response was observed for the majority (29/36 responders; 80·6%) of patients in the FAS [95% confidence interval (CI) 63·98–91·81%]. The lower limit of the one‐sided 97·5% CI for the proportion of responders was above the pre‐defined reference value of 60%, allowing the null hypothesis to be rejected and confirming the efficacy of IVIG 10% (Panzyga^®^). The response rate was very similar in the PP set (27/33 responders; 81·8%).

#### Secondary end points

**Figure 1:**
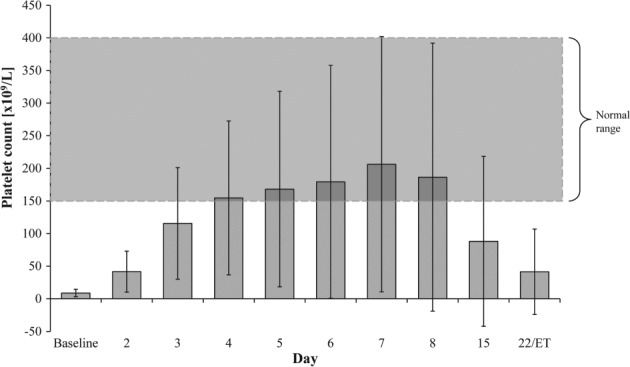
Platelet count over time in the full analysis set (*N* = 36). ET, early termination.

**Figure 2:**
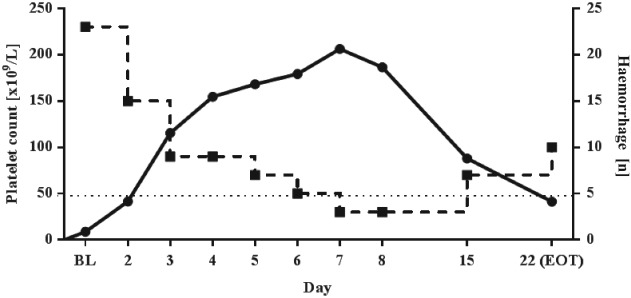
Change in mean platelet count versus all haemorrhages over time in the full analysis set (*N* = 36). Solid line = platelet count; thick dotted line = haemorrhage count; thin dotted line = complete response threshold (CR = platelet count ≥50 × 10^9^ L^−1^ within 8 days post‐treatment). BL, baseline; CR, clinical response; EOT, end of treatment.

The proportion of patients in the FAS with an alternative response or complete response were 66·7% and 50·0%, respectively, whereas 41·7% of patients were considered non‐responders. It is important to note that, with these EMA definitions, it is possible to achieve an alternative response while also being classified as a non‐responder (Table S1). The median time to response and response duration were 2 and 14 days, respectively. The mean maximum platelet count achieved in the 36 subjects was 237 × 10^9^ L^−1^. Changes in mean platelet counts over time are shown in Fig. 1 and Table S2. In 18 of the 23 patients (78·3%) who had bleeding at baseline, the haemorrhages completely resolved by day 8 (Table 2). The relationship between change in mean platelet count from baseline to day 8 and the number of patients who experienced haemorrhages at these time points are shown in Fig. 2. Most importantly, a highly significant increase in platelet count was observed mostly within 24 h after the first dose (Fig. 1), i.e. on day 2 [from 8·8 × 10^9^ L^−1^ (±5·76) to 41·6 × 10^9^ L^−1^ (±31·41); *P* < 0·0001].

**Table 2:** Proportion of patients experiencing haemorrhage and severity of haemorrhages over the study population in the full analysis set

Type of haemorrhage^2^	*N* (%)		
	Baseline (*n* = 36)	Day 8 (*n* = 34)^1^	Day 22/ET (*n* = 36)
Any	23 (63·9)	3 (8·8)	10 (27·8)
Minor	14 (38·9)	2 (5·9)	7 (19·4)
Mild	2 (5·6)	1 (2·9)	1 (2·8)
Moderate	7 (19·4)	0	1 (2·8)
Severe	0	0	1 (2·8)
Epistaxis	6 (16·7)	0	3 (8·3)
Minor	3 (8·3)	0	3 (8·3)
Mild	2 (5·6)	0	0
Moderate	1 (2·8)	0	0
Oral bleeding	9 (25·0)	1 (2·9)	5 (13·9)
Minor	5 (13·9)	0	3 (8·3)
Mild	0	1 (2·9)	1 (2·8)
Moderate	2 (5·6)	0	1 (2·8)
Severe	2 (5·6)	0	0
Skin bleeding	21 (58·3)	2 (5·9)	8 (22·2)
Minor	9 (25·0)	1 (2·9)	8 (22·2)
Mild	10 (27·8)	1 (2·9)	0
Moderate	2 (5·6)	0	0

ET, early termination.

1 Data missing for two patients.

2 Assessed sites of bleeding were epistaxis, oral and skin as the immediate sites; all other bleeding events were reported and graded as AEs.

### *Safety*

The type, frequency and severity of the TEAEs reported during the study are presented in Table 3. Thirty patients (75%) experienced at least one TEAE during the study, and a total of 122 TEAEs were recorded. Most of the 122 TEAEs reported were mild to moderate in severity, with a total of 15 classed as severe. The most frequent TEAE reported during the study was headache, reported in 17 (42·5%) patients, followed by pyrexia reported in 9 (22·5%) patients.

**Table 3:** Summary of adverse events in patients with immune thrombocytopenia (safety set; *N* = 40). Only individual adverse events occurring in ≥5·0% of patients are reported

	*n* (%)	Number of episodes
TEAEs	30 (75·0)	122
Headache	17 (42·5)	22
Pyrexia	9 (22·5)	9
Autoimmune thrombocytopenia	6 (15·0)	8
Nausea	6 (15·0)	6
Anaemia	5 (12·5)	7
Vomiting	4 (10·0)	4
Dizziness	3 (7·5)	4
Asthenia	2 (5·0)	3
Chills	2 (5·0)	3
Idiopathic thrombocytopenic purpura	2 (5·0)	2
TEAE severity		
Mild	15 (37·5)	85
Moderate	10 (25·0)	22
Severe	5 (12·5)	15
Related TEAEs^3^	23 (57·5)	58
Headache	13 (32·5)	17
Pyrexia	8 (20·0)	8
Nausea	5 (12·5)	5
Vomiting	4 (10·0)	4
Dizziness	2 (5·0)	2
Chills	2 (5·0)	3
Anaemia	2 (5·0)	2
SAEs	6 (15·0)	10
Related SAEs	1 (2·5)	1
Other significant AEs**1**	13 (32·5)	43
Non‐serious AEs	28 (70·0)	112
AEs leading to withdrawal from study	1 (2·5)	1
AEs leading to withdrawal of study drug	3 (7·5)	5
Death**2**	2 (5·0)	2
Infusional AEs within 72 h	24 (60·0)	71

AEs, adverse events; SAEs, serious adverse events; TEAEs, treatment‐emergent adverse events.

1 Definition of significant AE: non‐serious and dose changed or product withdrawn or other action or drug therapy started.

2 Deaths due to sepsis (*n* = 1) and cerebral haematoma (*n* = 1); both events were considered not related to study drug.

3 Possibly or probably related.

One patient (2·5%) withdrew from the study, and three patients (7·5%) experienced an AE that resulted in withdrawal of the study drug. Two patients (5·0%) died during the study. One patient, a 25‐year‐old Caucasian male, died 6 days after the first infusion due to severe intraparenchymal cerebral bleeding; it was subsequently found that the patient had two major protocol violations: the patient had Evans syndrome and had been receiving prohibited medication, mycophenolate mofetil. The second patient, a 57‐year‐old Caucasian male, died because of severe sepsis on study day 22; both deaths were assessed as unrelated to the study drug.

When the relationship to the study drug was analysed, there were 58 TEAEs reported in 23 patients (57·5%) that were considered probably or possibly related to IVIG 10% (Panzyga^®^) (Table 4); headache was the most commonly occurring related TEAE (13 patients with 17 episodes). Infusion‐related AEs within 72 h after start of infusion were observed in 24 patients (60·0%), with a total of 71 episodes; of these, 54 episodes were considered related to the study drug. Except for one patient, all patients were able to receive at least one infusion at the highest rate of 0·08 mL (kg min)^−1^. The most common infusion‐related AEs were headache and pyrexia; gastrointestinal disorders were also common.

**Table 4:** Number of patients with treatment‐emergent (possibly or probably) adverse events by MedDRA system organ class and preferred term (safety set; *N* = 40). Only individual adverse events occurring in ≥5·0% of patients are reported

	Causality *n* (%)		
	Probable	Possible	Total
Any TEAE	13 (32·5)	10 (25·0)	23 (57·5)
Nervous system disorders	7 (17·5)	8 (20·0)	15 (37·5)
Headache	6 (15·0)	7 (17·5)	13 (32·5)
Dizziness	1 (2·5)	1 (2·5)	2 (5·0)
General disorders and administration site conditions	4 (10·0)	7 (17·5)	11 (27·5)
Pyrexia	2 (5·0)	6 (15·0)	8 (20·0)
Asthenia	0	1 (2·5)	1 (2·5)
Chills	2 (5·0)	0 (0)	2 (5·0)
Blood and lymphatic system disorders	3 (7·5)	0 (0·0)	11 (27·5)
Anaemia	2 (5·0)	0	2 (5·0)
Gastrointestinal disorders	5 (12·5)	2 (5·0)	7 (17·5)
Nausea	4 (10·0)	1 (2·5)	5 (12·5)
Vomiting	3 (7·5)	1 (2·5)	4 (10·0)
Investigations	0	2 (5·0)	2 (5·0)

MedDRA, Medical Dictionary for Regulatory Activities; TEAE, treatment‐emergent adverse event.

The results of the laboratory tests and vital signs assessment demonstrated no significant safety concerns. In each of the haematology and chemistry parameters, changes to abnormal laboratory values considered clinically significant were reported in three and two patients, respectively. Laboratory abnormalities consistent with haemolysis were found in 6 of 40 patients (15·0%), and 1 patient had clinically evident haemolysis that was reported as a TEAE and considered to probably be related to the study drug. Fluctuations in vital signs were minor and within expected range for patients undergoing infusion. No changes in viral status were assessed during the study.

## DISCUSSION

The clinical significance of ITP is mainly reflected by bleeding and bleeding risk due to low platelet counts in affected patients. To date, treatment has generally been recommended for patients with bleeding, significant bleeding risks or platelet counts ≤20–30 × 10^9^ L^−1^. In fact, bleeding is not usually observed in patients with platelet counts ≥30 × 10^9^ L^−1^, and treatment is not generally required unless they are at risk of bleeding, such as during surgery (Provan *et al.,* 2010; Neunert *et al.,* 2011). Most studies that have addressed the use of IVIG in ITP were largely harmonised regarding safety and efficacy. The latter primarily focused on increasing platelet counts to ≥50 × 10^9^ L^−1^, rather than on the onset increase of platelet counts and/or compensated haemostasis (Colovic *et al.,* 2003; Varga *et al.,* 2006; Robak *et al.,* 2009; Robak *et al.,* 2010). However, successful prevention of bleeding and/or achievement of compensated haemostasis by increasing platelet count to ≥30 × 10^9^ L^−1^ remains of utmost importance. In the present study, 80·6% of patients treated with IVIG 10% (Panzyga^®^) showed quantitative (increase in platelet counts) and qualitative (bleeding control) responses within 8 days of treatment initiation. Furthermore, 22 of the patients treated (55%) demonstrated increased platelet counts to ≥30 × 10^9^ L^−1^ 1 day after the first IVIG infusion (1 g kg^−1^), and another 9 patients (22·5%) achieved platelet counts ≥30 × 10^9^ L^−1^ 2 days after the first IVIG infusion.

The safety, tolerability and overall response rate (defined as an increase in platelet counts to ≥50 × 10^9^ L^−1^) when using IVIG 10% (Panzyga^®^) following administration of 2 g kg^−1^ IVIG in patients with ITP are similar to those of previous studies with other IVIG 10% brands. In an international, multicentre, randomised, double‐blind, non‐inferiority trial of IVIG‐C vs solvent/detergent‐treated IVIG‐S/D in patients with ITP (platelet count at baseline ≤20 × 10^9^ L^−1^), response rates (defined as a platelet count of ≥50 × 10^9^ L^−1^ within 7 days of IVIG administration) were 90 and 83% with IVIG‐C and IVIG‐S/D, respectively (Bussel *et al.,* 2004). In three open‐label, multicentre studies of patients with ITP with platelet counts ≤20 × 10^9^ L^−1^, 80–83·3% of patients achieved a platelet count of ≥50 × 10^9^ L^−1^ within 6–7 days of treatment with Octagam^®^, Nanogam^®^ and Privigen^®^ (Robak, Salama *et al.,* 2009; Robak *et al.,* 2010; van der Meer *et al.,* 2011). In an open‐label study of Vigam‐S in patients with ITP with either a baseline platelet count of <20 × 10^9^ L^−1^ or >20 × 10^9^ L^−1^ with bleeding complications or requiring surgery, response was defined as an incremental increase of ≥30 × 10^9^ L^−1^, and 75% of the study population responded to treatment according to this criterion (Newland *et al.,* 2001). Similar to the results obtained in the present study, the median time to response in the aforementioned studies ranged from 2 to 4 days, whereas the duration of response ranged from 5 to 42 days (Newland *et al.,* 2001; Bussel *et al.,* 2004; Robak *et al.,* 2009; Robak *et al.,* 2010; van der Meer *et al.,* 2011). The range of peak platelet counts in previous studies (Newland *et al.,* 2001; Bussel *et al.,* 2004; Robak *et al.,* 2009; Robak *et al.,* 2010; van der Meer *et al.,* 2011) were also similar to the results obtained in the present cohort (∼160–273 × 10^9^ L^−1^). The most frequent AE associated with IVIG 10% (Panzyga^®^) was headache, as reported in a previous study and for other IVIGs (Varga *et al.,* 2006; Debes *et al.,* 2007; Robak *et al.,* 2010). Changes in laboratory parameters were as expected, with few patients reporting clinically significant changes. Haemolysis, observed in 15% of patients in our study, is commonly observed with IVIG preparations for this indication (Colovic *et al.,* 2003; Robak *et al.,* 2009; Robak *et al.,* 2010), and it has been postulated that patients with inflammatory conditions are at particular risk of developing haemolysis after receiving high doses of IVIG (Daw *et al.,* 2008).

The infusion protocol used in this study allowed for a rapid infusion of 1 g kg^−1^ Panzyga^®^. In contrast, for a IVIG 5% like Gammaplex with a maximum infusion rate of 0·08 mL (kg min)^−1^ (with ramp‐up phases of only 15 min duration, starting from 0·01 mL (kg min)^−1^, the infusion of 1 g kg^−1^ IVIG would take 4·7 h (282 min, similar to an infusion with Octagam^®^ 5%). This is approximately 1·5 h longer than the infusion rate seen with Panzyga^®^.

It should be noted that many of the patients in this study had severe chronic ITP (mean platelet count <8·8 × 10^9^ L^−1^) at baseline; this was thought to be predominantly due to the lack of availability of IVIG in a number of the study site countries (e.g. India).

One potential limitation of the study was that 22·5% of patients did not complete IVIG treatment for several reasons, including withdrawal of consent and occurrence of an AE. Strengths of the study include the study design, which adheres to the design proposed by the EMA for efficacy studies of IVIG preparations and allows the efficacy of IVIG 10% (Panzyga^®^) in patients with ITP to be established. It might be perceived that reducing the patient number from a target of 95 to 40 (a reduction in patient number that was agreed to following consultation with the US Food and Drug Administration) could have an impact on the results, but the data from the study and associated CIs for the primary end point conform to the originally defined success criteria and confirm the efficacy of IVIG 10% (Panzyga^®^).

## CONCLUSIONS

IVIG 10% (Panzyga^®^) is effective at rapidly increasing platelet counts in patients with ITP, with a response rate of >80% and a time to and duration of response similar to other IVIGs. Treatment with IVIG 10% (Panzyga^®^) was well tolerated, and there were no unexpected safety issues. These results suggest that IVIG 10% (Panzyga^®^) is an effective and safe treatment for patients with ITP.

## CONFLICT OF INTEREST

A. B. T. is an employee (Senior Global Clinical Project Manager) of Octapharma (Octapharma Pharmazeutika Produktionsges.m.b.H., Vienna, Austria). Dr S. W. was an employee (Head of Global Medical and Scientific Affairs) of Octapharma (Octapharma Pharmazeutika Produktionsges.m.b.H., Vienna, Austria) when the study was conducted. Currently, Dr S. W. is working as a Freelancer Medical Affairs and Clinical Study Management for Octapharma (Octapharma Pharmazeutika Produktionsges.m.b.H., Vienna, Austria). Dr A. S., Dr O. A. and Dr L. C. declare no conflict of interest.

## Supporting information


**Table S1.** European Medicines Agency definitions of response
**Table S2.** Platelet count (×10^9^ L^−1^) by study visit in the full analysis set (*N* = 36).
