# Supplementary material for: Efficacy and safety of a new intravenous immunoglobulin (Panzyga®) in chronic immune thrombocytopenia
Source: Transfus Med. 2019 Jan 27;29(1):48–54. doi: 10.1111/tme.12573 (PMC6850321; doi:10.1111/tme.12573)
Supplement: Supplementary file 1 — Table S1. European Medicines Agency definitions of response Table S2. Platelet count (×109 L−1) by study visit in the full analysis set (N = 36). [file TME-29-48-s001.docx]

# Supplementary tables

**Table S1.** European Medicines Agency definitions of response

*Committee for Medicinal Products for Human Use (CHMP): Guideline on the clinical investigation of human normal immunoglobulin for intravenous administration (IVIg) (EMA/CHMP/BPWP/94033/2007 rev. 2). 2010.* [*http://www.ema.europa.eu/docs/en_GB/document_library/Scientific_guideline/2009/10/WC500004766.pdf*](http://www.ema.europa.eu/docs/en_GB/document_library/Scientific_guideline/2009/10/WC500004766.pdf) *(Last accessed November 3, 2014).*

|  | **Definition** |
| --- | --- |
| AR | Increase in platelet count to ≥30x10^9^/L and to ≥2x baseline platelet count, confirmed on ≥2 separate occasions at least 7 days apart, and absence of bleeding |
| CR | Increase in platelet count to ≥100x10^9^/L, confirmed on ≥2 separate occasions at least 7 days apart, and absence of bleeding |
| Loss of AR/CR | AR/CR which subsequently deteriorated, to a platelet count of <30x10^9^/L/100x10^9^/L or to a level <2x the baseline count, or because of bleeding |
| Non-response | Platelet count of <30x10^9^/L or <2x increase in baseline platelet count, confirmed on 2 separate occasions ~1 day apart, or the presence of bleeding |
| AR, alternative response; CR, complete response. | |

**Table S2.** Platelet count (x10^9^/L) by study visit in the full analysis set (N=36).

| **Visit** | **N** | **Mean ± SD** | **Median (range)** |
| --- | --- | --- | --- |
| Baseline | 36 | 8.8 ± 5.76 | 8.5 (0–19) |
| Day 2 | 36 | 41.6 ± 31.41* | 38.5 (2–118) |
| Day 3 | 35 | 115.0 ± 85.58 | 102.0 (4–353) |
| Day 4 | 34 | 154.6 ± 118.04 | 126.5 (4–496) |
| Day 5 | 36 | 168.2 ± 149.89 | 131.7 (5–695) |
| Day 6 | 36 | 179.3 ± 178.64 | 136.3 (4–872) |
| Day 7 | 34 | 206.3 ± 195.75 | 175.0 (3–967) |
| Day 8 | 34 | 186.5 ± 205.44 | 153.0 (4–1067) |
| Day 15 | 34 | 88.1 ± 130.07 | 53.9 (3–685) |
| Day 22/ET | 36 | 41.4 ± 65.48 | 18.0 (1–295) |
| * 22 patients had platelet counts ≥30x10^9^/L. ET: early termination; SD: standard deviation. | | | |
